# The influence of preexisting coronary artery disease on long-term follow up and neurological outcome in patients receiving out of hospital extracorporeal membrane oxygenation^☆^

**DOI:** 10.1016/j.resplu.2025.101033

**Published:** 2025-07-14

**Authors:** Andrea Stadlbauer, Alois Philipp, Maik Foltan, Christian Stadlbauer, Simon Schopka, Christof Schmid, Andreas Keyser

**Affiliations:** Department of Cardiothoracic Surgery University Medical Center Regensburg, Germany

**Keywords:** ECPR, Coronary artery disease, Outcome

## Abstract

**Background:**

Pre-hospital extracorporeal cardiopulmonary resuscitation (ECPR) in out-of-hospital cardiac arrest is costly and resource-intensive. Low survival rates raise questions concerning efficacy of ECPR. We aimed to analyze survival and neurological outcome of these patients and the influence of underlying coronary artery disease as well as shockable heart rhythm leading to resuscitation.

**Methods:**

Retrospective analysis of our ECMO database revealed 94 patients receiving ECPR for out-of-hospital cardiac arrest from September 2009 to May 2023. After exclusion of patients with pulmonary embolism, drowning or intoxication as confounders, 58 patients remained. Patients were divided into 2 groups depending on underlying coronary artery disease and initial heart rhythm. Primary outcome was survival to discharge and long-term survival, secondary outcome was neurological capacity analyzed with the cerebral performance category score (CPC).

**Results:**

26 patients (44.8 %) survived to discharge; 6 patients died during a median follow-up time of 1057.5 days. There was no significant difference concerning survival to discharge between the groups. Numerically, more patients with shockable rhythm and without coronary artery disease survived. Kaplan-Meier analysis revealed a survival benefit for patients with shockable rhythm without coronary artery disease (*p* < 0.007). 92.3 % of survivors had a CPC-Score of 1. CPC Score did not differ between the groups.

**Conclusion:**

Though mortality in ECPR patients remains high with 55.2 %, long-term and neurological outcome with a CPC score of 1 is very good, especially of those with shockable rhythm and without coronary artery disease. Old age and duration of cardiopulmonary resuscitation pre-ECMO impair neurological outcome. Thus, on-site ECMO cannulation should be endorsed.

## Introduction

Out-of-hospital cardiac arrest (OHCA) is still associated with high mortality despite advancements in emergency medical care.^1^, ^2^ Survival to discharge rates remain below 10 %, with coronary artery disease (CAD) as predominant cause for cardiac arrest.^3^, ^4^ Initial rhythm causing cardiac arrest is known to influence outcome, with shockable rhythm being beneficial for survival.^5^ As survival outcomes remain suboptimal with Standard Advanced Cardiovascular Life Support (ACLS), guidelines recommend extracorporeal cardiopulmonary resuscitation (ECPR), but only in selected patients, when feasible, and when conventional CPR is failing.^6^ As time from start of CPR to extracorporeal membrane oxygenation (ECMO) initiation is crucial, some centers practice pre-hospital ECMO implementation, accepting increased costs, time, and resource demands.^7^ Efficacy of ECPR is a topic of ongoing debate, as studies yielded divergent results regarding patient outcomes,^8^, ^9^, ^10^, ^11^ and the question arises whether the benefit to the patient justifies the significant effort and expense that ECPR entails.

This study aimed to evaluate short-term and long-term survival of patients receiving on-site ECPR for OHCA, with neurological outcomes. Additionally, the study will examine the impact of CAD and the initial rhythm at the onset of resuscitation.

## Methods

### Study design and patient selection

**Fig. 1:**
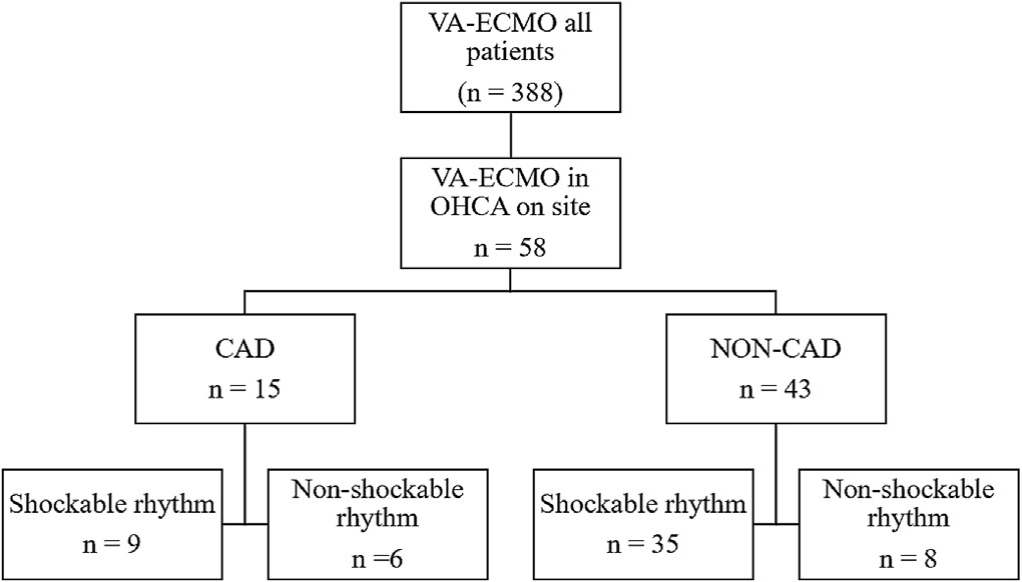
Flow-chart explaining the study design and patient selection.

A retrospective analysis of our institutional ECMO database was performed; all patients ≥ 18 years receiving veno-arterial (VA) ECMO due to OHCA between September 2009 to May 2023 were analyzed. To avoid biasing factors, patients with pulmonary embolism, drowning, or intoxication were excluded. Only patients who received ECPR due to a life threatening cardiac rhythm event were included and divided into patients with underlying coronary artery disease (CAD group) and those without (NON-CAD group). These groups were again divided according to initial rhythm at cardiac arrest (CAD-shockable vs CAD-non-shockable rhythm and NON-CAD-shockable vs NON-CAD-non-shockable rhythm; see Fig. 1).

This retrospective single-center cohort study analyzed patients who underwent out-of-hospital extracorporeal cardiopulmonary resuscitation (ECPR). Ethical approval was granted by the University of Regensburg Ethics Committee (Case No.: 24-3843-104); individual informed consent was waived due to the retrospective nature of the study.

Pre-implantation characteristics and outcome data—such as the presence of coronary artery disease (CAD) and the initial rhythm leading to CPR—were retrieved from electronic medical records. Relevant CAD was defined as either (1) known CAD with previous coronary intervention or (2) newly diagnosed CAD with ≥ 50 % stenosis on coronary angiography, regardless of whether an intervention was performed.

ECPR was defined as ongoing cardiopulmonary resuscitation without return of spontaneous circulation (ROSC), necessitating the initiation of veno-arterial extracorporeal membrane oxygenation (VA-ECMO). Inclusion and exclusion criteria for ECPR at our institution have been described in detail previously.^7^

Shockable rhythms were defined as ventricular fibrillation (VF) or pulseless ventricular tachycardia (VT), while non-shockable rhythms included pulseless electrical activity (PEA) and asystole.

All patients were cannulated in the prehospital setting (e.g., at home or at the scene) by our specialized mobile ECMO team, which includes experienced physicians, critical care nurses, and perfusionists. ECMO initiation and management were performed according to a standardized institutional protocol.^12^

Successful ECMO weaning was defined as survival after complete removal of ECMO support without the need for further mechanical circulatory support or heart transplantation, as established in prior studies.^13^, ^14^, ^15^

### Definition of outcome variables

The primary outcome measures were survival to discharge and long-term survival, defined as survival 6 months or more after discharge from hospital.

The secondary outcome variable was favorable neurological outcome at hospital discharge. The neurological assessment was performed using the Glasgow-Pittsburgh Cerebral Performance and Overall Performance Categories (CPC) score. Good neurological recovery was defined as a CPC score of 1 or 2.

### Statistical analysis

Statistical analysis was performed with IBM SPSS Statistics 25 (IBM Corp., Armonk, NY). For data collection before import into SPSS we used Excel for Windows (Microsoft Corp, Redmond, WA, USA).

Continuous data were presented as mean with standard deviation. Normal distribution was formally tested with the Shapiro-Wilks-test.

Categorical data were presented as frequencies and percentages.

Comparison of continuous variables was performed using the student’s *t*-test and the Mann-Whitney-*U* test depending on their normality, which was assessed by the Kolmogorov-Smirnov test.

Categorical variables were compared using Pearson’s chi-square-test or Fisher’s exact test, as appropriate. A bilateral p-value < 0.05 was considered statistically significant.

Regression analysis was performed to identify risk factors for the outcome variable ‘survival to discharge’ and ‘favourable neurological outcome’.

Kaplan-Meier-survival-analysis was used to estimate the proportion of survivors and visualized in survival curves.

Propensity-score-matching (PSM) was performed to rule out confounding factors.

## Results

### Study population

Retrospective analysis of our ECMO database revealed that out of 388 patients receiving ECPR between September 2009 and May 2023, 94 were cannulated due to OHCA and received pre-hospital ECMO implantation. 58 patients were resuscitated due to life threatening cardiac rhythm and included in this study.

Most patients were male (*n* = 41; 70.4 %). Mean age was 53.7 ± 14.1 years (median 55.1 years).

Median left-ventricular ejection fraction (LVEF) was 35.3 ± 16.7 % (min 0; max 65).

15 patients (25.9 %) had known or newly diagnosed CAD; 7 (12.1 %) showed new stenosis in coronary angiography and received an intervention.

Primary heart rhythm leading to ECPR was VF in 44 patients (75.9 %), the remaining patients presented with PEA (*n* = 8; 13.8 %) or asystole (*n* = 6; 12.1 %).

The time from start of CPR to initiation of ECMO was 49.3 ± 20.2 min (min 20; max 100 min).

Median duration of ECMO therapy was 3 ± 2 days (min 0; max 10 d).

Intensive-Care Unit (ICU) stay was 11.2 ± 13.1 days (min 0; max 85 d); overall hospital stay was 17.2 ± 27.3 days (min 0; max 193 d).

Patients’ characteristics are displayed in Table 1**.**

**Table 1:** Demographic data.

	**All patients** **(*n* = 58)**	**CAD** **(*n* = 15)**	**NON-CAD** **(*n* = 43)**	**p-value**
Age (years)	53.7 ± 14	60.1 ± 12.3	51.4 ± 14.2	**0.022**
Male	41 (70.7 %)	14 (93.3 %)	27 (62.8 %)	**0.025**
BMI (kg/m^2^)	27 ± 6	27 ± 5	32.3 ± 10	0.838
Pre-lactate (mmol/l)	11.2 ± 5.2	9.2 ± 3.8	14.1 ± 7.2	0.082
Pre-norepinephrine (µg/kg/min)	0.04 ± 0.1	0.09 ± 0.13	0.5 ± 0.5	0.238
Pre-epinephrine (µg/kg/min)	0.01 ± 0.06	0.05 ± 0.12	0.2 ± 0.3	0.076
Pre-MAP (mmHg)	37 ± 8	36 ± 7.6	44 ± 16	0.98
Pre-pH	7.2 ± 0.19	7.3 ± 0.18	7.2 ± 0.19	0.187
Peak NSE-level (µg/l)	143 ± 168	115 ± 131	153 ± 179	0.854
LVEF (%)	35.3 ± 16.7	31.4 ± 16.9	36.9 ± 16.6	0.31
Shockable rhythm	44 (75.8 %)	9 (60 %)	35 (81.4 %)	0.095
Duration of CPR (min)	49.3 ± 20.2	50 ± 18	58.9 ± 34.3	0.76
Average ECMO support (days)	3 ± 2	3.9 ± 2	5.8 ± 8.6	**0.014**
ICU stay (days)	11.2 ± 13.1	12.4 ± 9	9.5 ± 12.5	0.244
Overall hospital stay (days)	17.2 ± 27.3	19 ± 18	9.5 ± 12.5	0.233

BMI: body mass index; LVEF: left ventricular ejection fraction; MAP: mean arterial pressure; NSE: neuron-specific enolase; CPR: cardiopulmonary resuscitation; ICU: intensive-care unit.

To analyze the influence of preexisting CAD and initial heart rhythm on outcome, patients were divided into the CAD (*n* = 15; 25.9 %) and NON-CAD group (*n* = 43; 74.1 %). These groups were again divided into a shockable and non-shockable rhythm group (CAD-shockable: *n* = 9; 60 % vs. CAD-non-shockable: *n* = 6; 40 %; NON-CAD-shockable: *n* = 35; 81.4 % vs NO-CAD-non-shockable: *n* = 8; 18.6 %).

CAD patients were significantly older (CAD: 60.1 ± 12.3 years vs NON-CAD: 51.4 ± 14.2 years; *p* = 0.022). This age difference remained significant even when considering only those with a shockable rhythm (CAD-shockable: 64 ± 7 years vs. NON-CAD-shockable: 51 ± 15 years; *p* = 0.011).

CPR duration was longer in NON-CAD patients (CAD: 50 ± 18 min vs NON-CAD: 58.9 ± 34.3 min; *p* = 0.76). The numerical gap was even bigger when adjusted to shockable heart rhythm (CAD-shockable: 54 ± 19 min vs NON-CAD-shockable: 47.7 ± 12 min; *p* = 0.336).

### Primary outcome

Successful weaning from ECMO was possible in 26 patients (44.8 %; CAD: *n* = 7, 26.9 %; NON-CAD: *n* = 19; 73.1 %; *p* = 0.9). 26 patients (44.8 %) survived to discharged; most common cause of in-hospital death was post-anoxic brain injury (*n* = 26; 44.8 %; CAD: *n* = 6; NON-CAD: *n* = 20), three patients (5.2 %; CAD: *n* = 1; NON-CAD: *n* = 2) died of persistent low cardiac output, two patients (3.4 %; CAD: *n* = 1; NON-CAD: *n* = 1) died of multi-organ failure, one patient died from uncontrolled bleeding.

There was a higher proportion of survivors in the NON-CAD group (NON-CAD: 73.1 %, CAD: 26.9 %; *p* = 0.87), but without statistical significance. 22 of the survivors presented with initially shockable heart rhythm (shockable: 84.6 %, non-shockable: 15.4 %; *p* = 0.16).

Survivors without CAD had shockable rhythm more often than survivors with CAD (NON-CAD-shockable: 81.8 %, CAD-shockable: 18.2 %; *p* = 0.71).

All survivors were alive 30 days after hospital discharge (100 %). 25 patients were alive after 6 months or more. Median follow up was 1057.5 days (Min 55 d; Max 3881 d).

Six patients (10.2 %) died after a median follow up of 957 days.

**Fig. 2:**
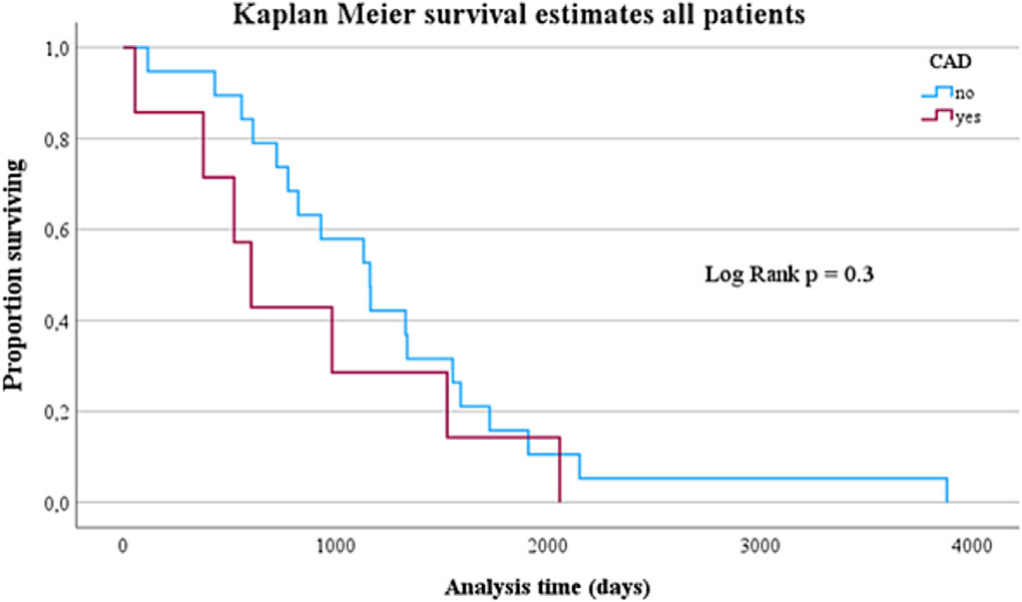
Kaplan-Meier estimation of survival of all patients (*n* = 26) over follow-up time: comparison with and without CAD.

Chances of long-term survival did not differ between NON-CAD and CAD patients (Fig. 2**;** *p* = 0.3).

**Fig. 3:**
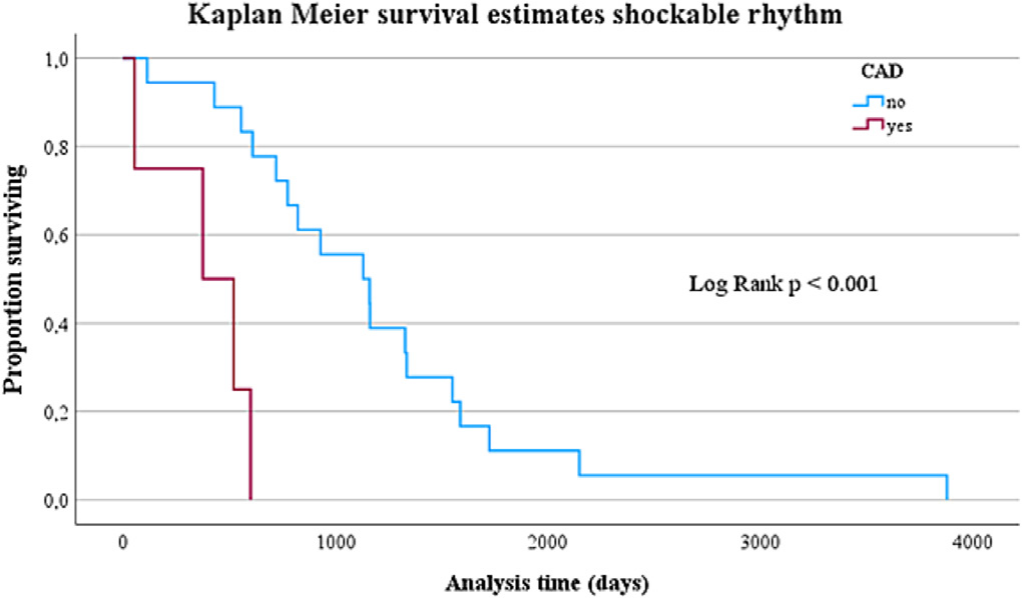
Kaplan-Meier estimation of survival over follow-up time in patients with shockable rhythm (*n* = 22): comparison with and without CAD.

The long-term survival of patients with shockable heart rhythm receiving ECMO revealed a survival benefit for those without CAD (Fig. 3**;** *p* < 0.001).

### Secondary outcome

CPC 1 was reached by 24 patients (92.3 %), one patient had CPC 2 (3.8 %) and one CPC 4 (3.8 %).

Favorable neurological outcome was similar between CAD and NON-CAD patients (CAD: 100 %, NON-CAD: 94.7 %; *p* = 0.54), likewise in patients with shockable or non-shockable heart rhythm (shockable: 95.5 %, non-shockable: 100 %; *p* = 0.66).

CPC Score of patients with shockable heart rhythm and CAD was not impaired when compared with patients without CAD (CAD: 94.4 %, NON-CAD: 100 %; *p* = 0.63).

### Regression analysis survival to discharge

Univariate Regression analysis revealed a higher LVEF (≥ 45 %; *p* ≤ 0.001), longer ICU and overall hospital stay (*p* ≤ 0.001), high hemoglobin levels prior to ECMO (*p* = 0.04), higher central venous oxygen saturation prior to ECMO (*p* = 0.03), higher prothrombin time prior to ECMO (*p* = 0.015) and MAP ≥ 60 mmHg on day one after ECMO start (*p* = 0.01) as factors positively impacting survival. Prolonged CPR prior to ECMO (*p* ≤ 0.02), elevated lactate levels prior to ECMO (*p* = 0.02) and the day after (*p* = 0.05), more acidic pH levels (*p* = 0.03), the need for escalated ventilation parameters (FiO2: *p* = 0.03; peak pressure: *p* = 0.007), elevated partial thromboplastin time (aPTT; *p* = 0.022), d-dimer levels (*p* = 0.03), elevated tumor necrosis factor-α (TNF-α; *p* = 0.002) and a high peak neuron-specific enolase level (NSE; *p* = 0.006) negatively influenced survival.

In multivariate analysis, none of these remained significant.

### Regression analysis CPC score

Increasing age (*p* = 0.014), longer CPR duration (*p* = 0.002), need for higher vasopressor dose one day after ECMO initiation (*p* = 0.05) and elevated levels of d-dimers (*p* = 0.04), interleukin 2 (*p* = 0.04) and interleukin 8 (*p* = 0.022) prior to ECMO were significant risk factors for a higher CPC Score at discharge in univariate analysis. In the multivariate analysis, only old age (*p* = 0.24) and CPR duration (*p* = 0.008) remained with unfavorable neurological outcome increasing significantly when CPR duration lasted ≥ 50 min.

### Outcome variables without cerebrovascular death

After excluding patients who had died due to cerebral hypoxia as confounders, 32 patients remained for this analysis (CAD: *n* = 9, 28.1 %; NON-CAD: *n* = 23, 71.9 %) (Table 2).

**Table 2:** Demographic data patients without cerebrovascular death.

	**All patients** **(*n* = 32)**	**CAD** **(*n* = 9)**	**NON-CAD** **(*n* = 23)**	**p-value**
Age (years)	57 ± 12	60.1 ± 12.3	54.3 ± 12.1	**0.022**
Male	24 (75 %)	9 (37.5 %)	15 (65.2 %)	**0.041**
BMI (kg/m^2^)	27.7 ± 6.6	27.8 ± 5	27.7 ± 7	0.767
Pre-lactate (mmol/l)	9.4 ± 5.4	8.1 ± 3.5	9.9 ± 6	0.55
Pre-norepinephrine (µg/kg/min)	0.05 ± 0.1	0.08 ± 0.13	0.04 ± 0.1	0.33
Pre-epinephrine (µg/kg/min)	0.005 ± 0.03	0.02 ± 0.05	0.02 ± 0.03	0.11
Pre-MAP (mmHg)	38 ± 7	38 ± 6	38 ± 7	0.91
Pre-pH	7.3 ± 0.1	7.4 ± 0.1	7.3 ± 0.1	0.55
Peak NSE-level (µg/l)	48.6 ± 37.6	41 ± 8	51 ± 43	0.98
LVEF (%)	40.5 ± 14	35 ± 17	43 ± 13.3	0.25
Shockable rhythm	26 (81.2 %)	7 (26.9 %)	19 (73.1 %)	0.095
Duration of CPR (min)	40.5 ± 15.3	44.6 ± 17.3	39 ± 14.5	0.4
Average ECMO support (days)	3 ± 2	3.7 ± 2.4	2.7 ± 2	0.16
ICU stay (days)	16.7 ± 15.1	14.7 ± 10	17.5 ± 16.8	0.96
Overall hospital stay (days)	27 ± 33	24 ± 20	28 ± 37.6	0.95
Survival to discharge	25 (78.1 %)	6 (18.8 %)	19 (59.4 %)	0.33

BMI: body mass index; LVEF: left ventricular ejection fraction; MAP: mean arterial pressure; NSE: neuron-specific enolase; CPR: cardiopulmonary resuscitation; ICU: intensive-care unit.

Again, patients with CAD were significantly older (*p* = 0.022). No significant difference remained in ECMO-support duration (CAD: 3.7 ± 2.4 days vs NON-CAD: 2.7 ± 2 days; *p* = 0.16).

25 patients were weaned successfully from ECMO and survived to discharge (CAD: 6 (18.8 %) vs NON-CAD: 19 (59.4 %); *p* = 0.33), with a higher proportion of survivors in the NON-CAD group.

Survival analysis revealed no significant difference in survival between CAD and NON– CAD patients. The presence of a shockable rhythm did not influence the survival outcomes in either group (Supplementary material, Figure A and B).

Only one patient exhibited bad neurological outcome with a CPC Score of 4, while the other survivors didn’t differ in CPC Score (CAD: 1 vs NON-CAD: 1.2 ± 0.7; *p* = 0.42).

Regression analysis revealed several predictors for survival to discharged (Supplementary Material, Table 3), but none of them remained significant in multivariate analysis.

Linear regression analysis demonstrated an association between the CPR duration, vasopressor dosage on day one after ECMO initiation, venous pH, minute ventilation and peak airway pressure prior to ECMO, as well as IL-2 and IL-8 levels, with the CPC score. In multivariate analysis, none remained statistically significant.

### Propensity-score-matching

To further eliminate confounders, a propensity score matching was performed, adjusted for age, sex, shockable heart rhythm, CPR duration and LVEF. The final cohort consisted of 30 patients, with 15 in the CAD group and 15 in the NON-CAD group. These groups were compared based on demographic data, survival curves, and neurological outcome.

The groups differed significantly in ECMO support (CAD: 3.9 ± 2.1 days vs NON-CAD: 2.5 ± 2.5 days; *p* = 0.029), lactate levels pre-ECMO (CAD: 9.3 ± 3.8 mmol/L vs NON-CAD: 13.1 ± 5 mmol/L; *p* = 0.026), CRP levels on day one (CAD: 17.6 ± 17.3 mg/L vs NON-CAD: 48 ± 33 mg/L; *p* = 0.025) and cerebral In-Vivo-Optical-Spectoscopy values (INVOS) pre ECMO (CAD: 45 ± 13 vs NON-CAD: 32 ± 16; *p* = 0.049).

**Fig. 4:**
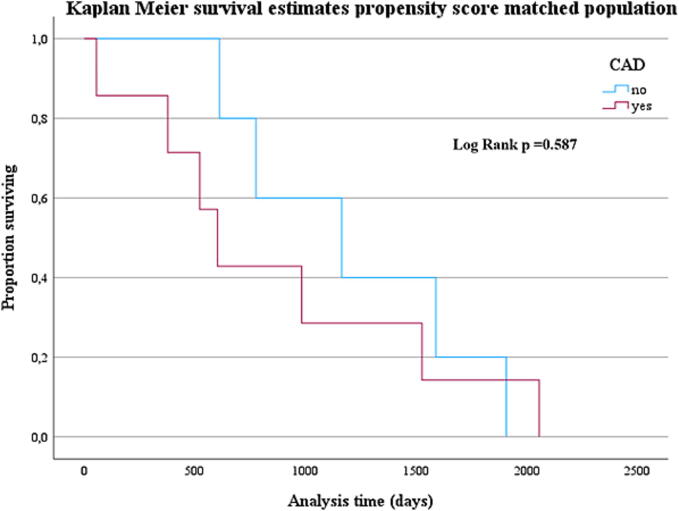
Kaplan-Meier estimation of survival over follow-up time propensity score matched population: comparison with and without CAD.

Survival to discharge was 40 % (12 patients) and did not differ significantly between the groups (Fig. 4).

**Fig. 5:**
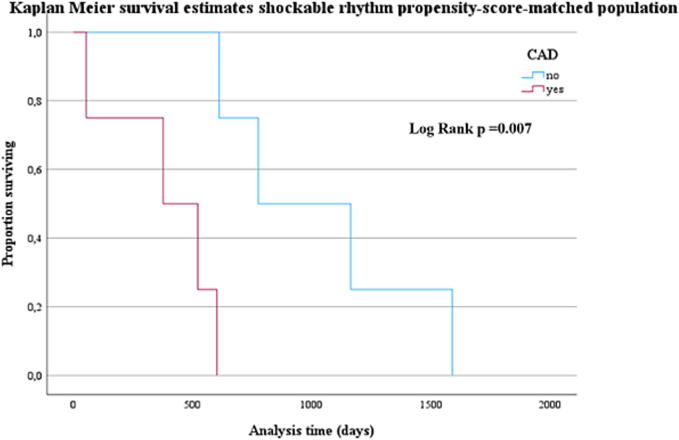
Kaplan-Meier estimation of survival over follow-up time in propensity score matched cohort with shockable rhythm: comparison with and without CAD.

NON-CAD patients with shockable heart rhythm still showed significantly better survival compared to those with CAD (Fig. 5).

Regression analysis for survival to discharge demonstrated a positive correlation for EF and ICU stay and a negative correlation prolonged CPR duration, elevated D-Dimer levels, elevated IL-6 levels prior to ECMO and elevated TNFα levels one day after ECMO initiation.

All survivors exhibited good neurology at discharge, without significant difference in CPC Scale between CAD and NON-CAD patients.

## Discussion

To our knowledge, this study is the first to analyze short-, long-term and neurological outcome of patients receiving pre-hospital ECPR in OHCA with special emphasis on the impact of underlying CAD and initial shockable heart rhythm.

Survival to discharge was 44.8 %. There was no statistically significant difference in terms of survival concerning CAD and underlying heart rhythm, but survivors had a higher proportion of non-CAD and shockable rhythms. In patients with shockable rhythm, survival differences by CAD status were not statistically significant. The absence of statistical significance, despite clear numerical differences, is most likely attributable to the limited sample size of patients.

Kaplan-Meier survival analysis showed no difference in long-term survival concerning underlying CAD in all patients, but a clear survival benefit for patients with initially shockable rhythm and no proof of CAD. Patients with CAD and shockable heart rhythm were significantly older and had a shorter CPR duration, albeit not statistically significant. There was a high proportion of individuals dying due to cerebrovascular hypoxia, which might be a potential bias.

Kaplan-Meier analysis without individuals dying due to cerebrovascular injury, showed no significant differences in terms of survival between CAD and NON-CAD patients. While in this cohort, CPR duration did not differ significantly between the groups, the huge age-gap between CAD and NON-CAD patients remained.

The observed decline of the Kaplan-Meier survival curve to zero reflects the end of the follow-up period rather than an abrupt mortality event, and should not be misinterpreted as a plotting artifact.

To further rule out potential confounders, propensity-score matching in respect to age, gender, shockable heart rhythm, CPR duration and LVEF was performed.

In this matched population, survival to discharge rate was still 40 % and did not differ between CAD and NON-CAD patients (see Fig. 4). When only considering those with shockable heart rhythm, the survival benefit for NON-CAD patients remained (Fig. 5).

One might assume that patients with CAD receiving ECPR would have improved survival rates due to the treatability of the underlying condition. Interestingly, in our cohort, the presence of treatable CAD did not confer a survival advantage. This is most likely due to the small sample size and cannot be generalized for larger cohorts.

Survival rate in our cohort was rather high compared to the 8–31 % reported in the literature.^10^, ^12^, ^13^, ^14^ The ARREST trial showed similar survival rates in their ECPR cohort with 43 %.^15^ To date, few studies analyze the influence of CAD on survival in OHCA patients receiving ECPR. Lamhaut investigated the clinical and angiographic characteristics of OHCA patients receiving ECPR. They found severe CAD, with a high rate of multiple vessel disease and proximal lesions, especially in patients with shockable rhythm. The authors could not prove any survival difference concerning CAD or initial rhythm.^16^

Consistent with our results, a clear survival advantage for ECPR-patients with an initially shockable rhythm exists in literature.^5^, ^17^ While some authors concluded non-shockable rhythm as a contraindication for ECPR, other authors have achieved comparable neurological outcomes despite asystole or PEA.^18^, ^19^, ^20^

All survivors had a favorable neurological outcome in our group, independent of underlying CAD or initial heart rhythm. Looking at the PSM cohort, all of the 12 survivors to discharge exhibited good neurological outcome. However, the CPC score is only assessed in survivors after hospital discharge and is not recorded in patients who die during hospitalization, which represents a potential source of ascertainment bias.

Old age and longer duration of CPR prior to ECMO were independent risk factors for poor neurological outcome.

Several *meta*-analyses comparing neurological outcome of ECPR versus conventional CPR (CCPR) exist: Gomes compared the three big randomized controlled trials (PRAGUE OHCA, INCEPTION, ARREST) and found ECPR associated with a non-statistically significant higher survival rate with favorable neurological outcome at hospital discharge and after six months.^21^ Low proved ECPR to be superior to CCPR with reduced in-hospital mortality and improved long-term neurological outcome.^8^ A *meta*-analysis examining studies addressing ECPR in OHCA patients, revealed a mean survival-to-discharge rate of 24 %, with 18 % favorable neurological outcome.^22^ Most studies included have only a low or no proportion of pre-hospital ECPR patients, and show low-flow times to ECPR initiation with a mean duration of 75 min.^8^, ^17^, ^22^ Mean duration of CPR prior to ECMO initiation was 49.3 ± 20.2 min in our cohort. Our mobile ECMO team operates day and night, alarmed directly by the integrated emergency services control center in case of any OHCA. If the patient is suitable for ECPR, our team immediately moves to the scene of accident with the necessary equipment by ground or air to cannulate the patient on site. This highly standardized process results in shorter low-flow times, explaining our high survival rate with good neurological outcome. Long duration of CPR prior to ECMO is a known risk factor for impaired neurological outcome in our and in many previous studies. While our patients had worse outcome if CPR duration exceeded 50 min, Yukawa specified the cut-off at 40 min^23^; Tran et al found decreasing favorable neurological outcome with every 10 min of longer CPR duration (OR 1.41; 95 %-CI 1.17–1.69), as well as old age as a factor negatively impacting functional status.^5^ By rapidly restoring organ perfusion, ECPR minimizes low-flow time, decreasing the risk of multi-organ failure, cardiovascular instability, and brain injury.^24^, ^25^ Old age has already been proven to be a risk factor negatively impacting outcome in ECMO patients in general. As older patients are more likely to have comorbidities like CAD, disrupted perfusion and organ hypoperfusion with ischemia is more likely.^5^, ^26^

In our study, underlying CAD or primary rhythm at cardiac arrest did not impact neurological outcome. So far, no study analyzed the impact of CAD on neurological outcome in patients receiving ECPR, though many have dealt with the influence of the primary heart rhythm at cardiac arrest. Inoue, Yannopoulos and Lunz et al found initially shockable rhythm as independent predictor of favorable neurological outcome.^2^, ^15^, ^17^ Two Japanese show comparable neurological outcome and good neurological outcome of 14.3 % for patients with PEA.^19^, ^20^ Both studies had a high proportion of patients with reversible reasons for cardiac arrest in the non-shockable group (e.g. pulmonary embolism, acute coronary syndrome), making ROSC and better outcome more likely.

Several limitations must be acknowledged. First, the sample size was relatively small, especially after propensity score matching. The data collection relied on retrospective data acquisition of a single center conducted in a specific geographical region, which may limit the generalizability of the findings. Future research should consider longitudinal approaches and larger multi-center samples to further validate the results.

## Conclusion

Despite the overall high mortality associated with ECPR in out-of-hospital cardiac arrest (OHCA), our study demonstrated a survival rate of 44.8 %, with 96.2 % of survivors achieving favorable neurological outcomes. Patients with shockable initial rhythms and without underlying coronary artery disease (CAD) showed a numerically higher survival, suggesting these factors may be associated with improved outcomes. Although these differences did not reach statistical significance, they highlight the importance of initial rhythm and cardiac pathology in patient selection and prognostication. The implementation of pre-hospital ECPR in a well-organized system has the potential to markedly improve outcomes for OHCA patients. However, further trials on prehospital cannulation are needed to further investigate the potential benefit for the patient. Out-of-hospital ECMO implantation should be reserved for highly experienced centers, given the complexity and risks associated with this procedure.

## CRediT authorship contribution statement

**Andrea Stadlbauer:** Writing – review & editing, Writing – original draft, Supervision, Project administration, Methodology, Investigation, Formal analysis, Data curation, Conceptualization. **Alois Philipp:** Data curation, Conceptualization. **Maik Foltan:** Data curation, Conceptualization. **Christian Stadlbauer:** Writing – review & editing. **Simon Schopka:** Writing – review & editing. **Christof Schmid:** Writing – review & editing. **Andreas Keyser:** Writing – review & editing, Writing – original draft, Project administration, Methodology, Investigation, Formal analysis, Data curation, Conceptualization.

## Declaration of competing interest

The authors declare that they have no known competing financial interests or personal relationships that could have appeared to influence the work reported in this paper.
