# Supplementary material for: The influence of preexisting coronary artery disease on long-term follow up and neurological outcome in patients receiving out of hospital extracorporeal membrane oxygenation
Source: Resusc Plus. 2025 Jul 14;25:101033. doi: 10.1016/j.resplu.2025.101033 (PMC12309585; doi:10.1016/j.resplu.2025.101033)

Supplementary Materials

**Table 3 Univariate regression analysis for predictors of survival to discharge (no cerebrovascular death)**

|  | **OR** | **95%-CI** | **p-value** |
| --- | --- | --- | --- |
| EF | 1.149 | 1.036–1.275 | **0.009** |
| Hospital stay | 1.344 | 1.066-1.695 | **0.012** |
| ICU stay | 1.321 | 1.076-1.622 | **0.008** |
| Lactate day 1 | 0.967 | 0.950-0.996 | **0.024** |
| MAP day 1 | 1.324 | 1.023-1.638 | **0.032** |
| Pre-hemoglobin | 1.947 | 1.068-3.550 | **0.030** |
| Peak-pressure day 1 | 0.540 | 0.298-0.980 | **0.043** |
| Pre-prothrombin | 1.096 | 1.025-1.172 | **0.007** |
| Pre-INR | 0.390 | 0.194-0.787 | **0.009** |
| Pre-platelets | 1.022 | 1.002-1.042 | **0.032** |
| Pre-TNFα | 0.874 | 0.765-0.998 | **0.046** |
| TNFα day 1 | 0.760 | 0.586-0.985 | **0.038** |
| PRB | 0.688 | 0.503-0.942 | **0.020** |
| FFP | 0.714 | 0.551-0.925 | **0.011** |

EF: ejection fraction; FFP: fresh frozen plasma; ICU: intensive care unit; INR: international normalized ratio; MAP: mean arterial pressure; PRB: packed red bloodcells, TNFα: tumor-necrosis-factor alpha

**Figure A** Kaplan-Meier estimation of survival over follow-up time in patients without cerebrovascular death: comparison with and without CAD
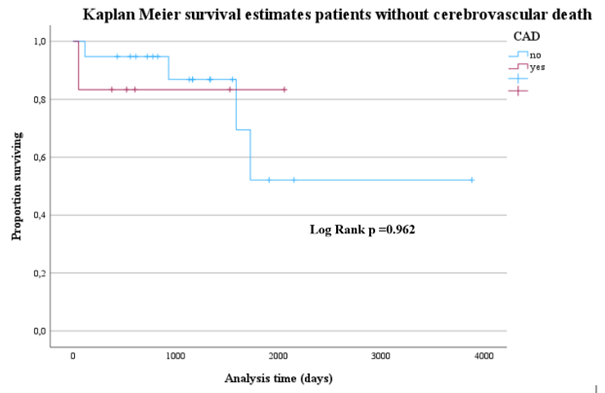


**Figure B** Kaplan-Meier estimation of survival over follow-up time in patients without cerebrovascular death and with shockable rhythm: comparison with and without CAD
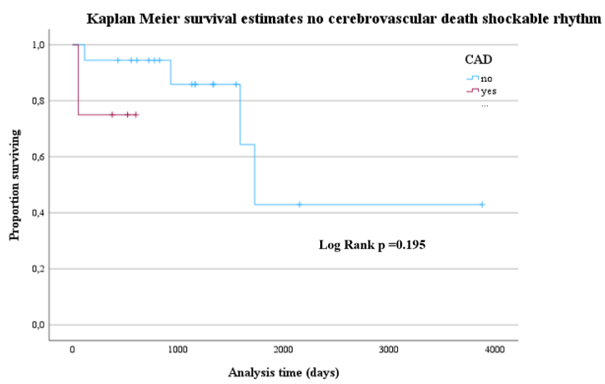

Supplement: Supplementary Data 1 [file mmc1.docx]
